# Computational Short Tandem Repeat Genotyping Reveals Clinically Relevant Expansions in a Large Turkish Neurodegeneration Disease Cohort †

**DOI:** 10.3390/ijms27104345

**Published:** 2026-05-13

**Authors:** Zakhiriddin Khojakulov, Robin J. Palvadeau, Müge Kovancılar-Koç, Irmak Atay, Irmak Şahbaz, Şeyma Tekgül, Ayça Şahin, Esmer Zeynep Duru Badakal, Tuğçe Gül-Demirkale, Vildan Çiftçi, Elif Bayraktar, Ceren Tunca, Natalia Smolina, Fulya Akçimen, Ayşe Nazlı Başak

**Affiliations:** 1 Neurodegeneration Research Laboratory (NDAL), Research Center for Translational Medicine (KUTTAM), School of Medicine, Koç University, 34010 Istanbul, Turkey; zkhojakulov20@ku.edu.tr (Z.K.); rpalvadeau@ku.edu.tr (R.J.P.); mugekoc@ku.edu.tr (M.K.-K.); iratay@ku.edu.tr (I.A.); esahbaz@ku.edu.tr (I.Ş.); stekgul@ku.edu.tr (Ş.T.); asahin15@ku.edu.tr (A.Ş.); ebadakal21@ku.edu.tr (E.Z.D.B.); tdemirkale@ku.edu.tr (T.G.-D.); vciftci@ku.edu.tr (V.Ç.); elbayraktar@ku.edu.tr (E.B.); cetunca@ku.edu.tr (C.T.); nsmolina@ku.edu.tr (N.S.); 2 Laboratory of Neurogenetics, National Institute on Aging, National Institutes of Health, Bethesda, MD 20892, USA; fulya.akcimen@nih.gov

**Keywords:** neurodegenerative diseases, computational genotyping, ExpansionHunter, short tandem repeats, STR, NGS

## Abstract

Short tandem repeat (STR) expansions are a major cause of neurodegenerative disorders; however, their genetic and clinical heterogeneity complicates diagnosis. STR detection remains limited in routine short-read next-generation sequencing (NGS) workflows. We evaluated the diagnostic yield and clinical utility of computational STR genotyping in a large Turkish neurodegenerative disease cohort. ExpansionHunter was applied to NGS data from 3150 patients and 146 controls, targeting 15 disease-associated STR loci. To improve genotyping of poorly captured exonic regions in exome data, the default locus coverage threshold was reduced from 10× to 3×. Candidate expansions were visually inspected using REViewer and validated by conventional molecular methods. Computational analysis detected 28 pathogenic and 160 intermediate expansions. Of these, 23 were confirmed as pathogenic, and eight initially classified as intermediate were reclassified as pathogenic after conventional validation, resulting in 31 pathogenic cases across 28 families: *HTT* (*n* = 8), *ATXN2* (*n* = 5), *ATXN1* (*n* = 4), *DMPK* (*n* = 3), *PABPN1* (*n* = 3), *TBP* (*n* = 2), and single cases in *AR*, *ATN1*, and *CACNA1A*. Lowering the coverage threshold markedly increased genotyping rates at low-coverage loci in exome data, particularly in *ATXN2*. Genetic findings were largely consistent with clinical pre-diagnosis and the additional diagnostic yield was 0.95%. These findings support integrating STR analysis into routine neurogenetic diagnostics.

## 1. Introduction

The human genome contains over one million tandem repeat loci, accounting for approximately 8% of the genome and contributing significantly to genomic variation. These loci are inherently unstable and exhibit high mutation rates, leading to substantial genetic variation. Among them, short tandem repeats (STRs), defined as repeating motifs of 2–6 base pairs, have been implicated in a wide range of diseases; over 50 loci are known to be associated with human disorders, particularly neurological conditions [1,2].

Repeat expansion disorders affect an estimated 1 in 3000 individuals [3]. These diseases are genetically and clinically heterogeneous, complicating genotype–phenotype correlations. Among the most commonly implicated genes are *ATXN1*, *ATXN2*, *ATXN3*, *ATXN7*, *ATN1* (atrophin-1), *CACNA1A*, *PPP2R2B*, and *TBP*, where (CAG/CTG)_n_ expansions are associated with different subtypes of spinocerebellar ataxia (SCA) [4]. Expansions in *ATXN2* and *ATXN3* extend beyond ataxia: mildly elevated pathogenic *ATXN2* repeats have been reported in familial Parkinsonism [5], and intermediate-range alleles are associated with increased risk of amyotrophic lateral sclerosis (ALS) [6]. In contrast, *ATXN3* expansions have been implicated in Parkinson’s disease (PD) [7]. Pathogenic *HTT* (huntingtin) repeat expansion has also been reported in patients with atypical motor symptoms at onset, including PD, dystonia, ataxia, ALS, and frontotemporal dementia (FTD) patients [8,9].

Taken together, these observations demonstrate how repeat expansions transcend traditional disease boundaries and exhibit variation across diverse populations. This heterogeneity highlights the need for robust and scalable genotyping methods, since standard short-read next-generation sequencing (NGS) pipelines often fail to detect STRs. Specialized tools, such as ExpansionHunter (EH), have been developed to estimate repeat sizes from short-read data, enabling the reliable detection of clinically relevant expansions [1,3,8,10,11,12,13,14,15,16].

In this study, we applied EH v5 to a large Turkish cohort of 3296 samples, including 3150 patients with movement and neuromuscular disorders and 146 matched controls. Although in silico genotyping approaches allow interrogation of numerous disease-associated STR loci, we restricted our analysis to 15 repeats that are clinically relevant to the disorders represented in our cohort and sufficiently captured by exome data. By systematically screening these loci, we identified 30 pathogenic expanded alleles in individuals presenting both canonical and atypical phenotypes. These findings underscore the value of systematic, large-scale STR screening for refining genotype–phenotype relationships across neurodegenerative and neuromuscular disorders.

## 2. Results

### 2.1. Study Overview

**Figure 1:**
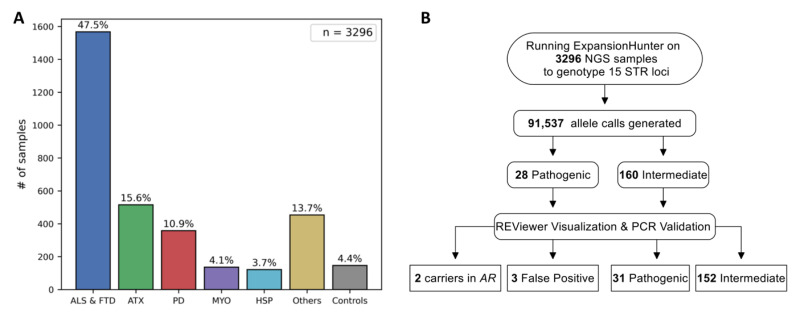
Phenotypes of the study cohort and overview of the workflow. (**A**) Disease distribution of the cohort. The bar labeled “Others” represents patients diagnosed with Alzheimer’s disease, leukodystrophy, spinal muscular atrophy, neurodegeneration with brain iron accumulation and other rare inherited disorders. (**B**) Schematic representation of workflow. PCR-based conventional validation led to the reclassification of eight intermediate expansions as pathogenic. ALS: amyotrophic lateral sclerosis, FTD: frontotemporal dementia, HSP: hereditary spastic paraplegia, MYO: myopathy, PD: Parkinson’s disease.

The study cohort comprised 3296 individuals, including 3150 patients with neurodegenerative and/or neuromuscular disorders and 146 healthy controls (Figure 1A). Of these, 1056 underwent genome (including controls), and 2240 exome sequencing; both were performed using heterogeneous sequencing kits (Supplementary Table S1). Fifteen disease-associated STR expansions, which are clinically relevant to the disorders represented in this cohort (Supplementary Table S2), were genotyped using EH v5. The *C9orf72* repeat expansion locus was excluded, as ALS and FTD patients were routinely prescreened for pathogenic expansions prior to NGS (Supplementary Figure S3). Coverage at these loci was markedly variable in exome sequencing compared with genome sequencing, primarily due to differences in capture kits (Supplementary Figures S1 and S2). Variants flagged as “LowDepth” in EH output were not included in downstream analysis (Figure 1B).

**Figure 2:**
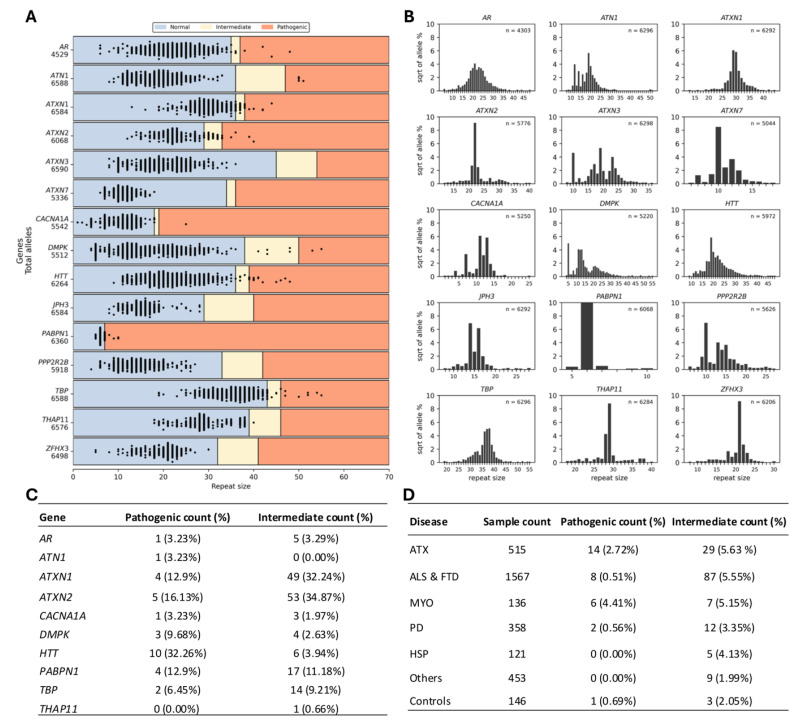
Repeat size distributions. (**A**) Swim lane plot showing the repeat sizes of STRs genotyped by EH. Samples are represented by two dots corresponding to two alleles, except for males at the *AR* locus. The total number of alleles per locus is displayed below each gene. (**B**) Square root–transformed percentage distribution of repeat sizes across 15 loci in patients. (**C**) Summary of locus-specific and (**D**) disease-category distribution of pathogenic and intermediate expansions following PCR refinement. ALS: amyotrophic lateral sclerosis, ATX: ataxia, FTD: frontotemporal dementia, MYO: myopathy, PD: Parkinson’s disease.

Since each individual has two alleles per locus, except for *AR* (androgen receptor) in males, a total of 91,537 allele calls were generated across the entire cohort. Figure 2A illustrates the repeat size distribution across the entire cohort based on molecular categorization (normal, intermediate, pathogenic), whereas Figure 2B presents the allele frequency distributions across loci in patients. The corresponding distribution in controls is shown in Supplementary Figure S4. Based on the figures, STR sizes in five loci (*ATXN2*, *ATXN7*, *PABPN1* (poly(A) binding protein nuclear 1), *THAP11*, and *ZFHX3*) exhibited marked allelic homogeneity, with a single repeat size accounting for 60% to over 90% of observed alleles, in contrast to the greater variability observed at the remaining loci.

### 2.2. Clinically Relevant Repeat Expansions

In this cohort, 14 pathogenic repeat expansions in 10 families had been previously identified using conventional diagnostic methods. EH reports both genotype estimates and corresponding confidence intervals, and all previously identified pathogenic expansions were correctly detected using their exome data. In each case, PCR-based repeat sizes fell within or were consistent with the reported genotype confidence intervals, providing internal validation of the in silico approach (Supplementary Table S3).

In addition to these previously identified cases, EH detected new 28 pathogenic and 160 intermediate repeat expansions across multiple loci, yielding a total of 188 potentially clinically relevant alleles across 183 individuals. Among these, three individuals harbored two intermediate expansions at distinct loci, and two individuals carried same-sized intermediate expansions in the *DMPK* (dystrophia myotonica protein kinase) locus.

Given that *AR*-associated repeat expansion disorders are inherited in an X-linked recessive manner, males require one pathogenic allele for disease manifestation, whereas females are typically asymptomatic carriers unless harboring two expanded alleles [1]. In our cohort, two pathogenic *AR* expansions were identified in females consistent with carrier status and were therefore not included in validation analyses. In addition, REViewer visualization revealed three potential false-positive calls at the *AR* (in one male), *ATXN1*, and *TBP* loci, due to insufficient reads supporting the expanded alleles or gaps in the alignment structure. (Supplementary Figures S5–S7). PCR validation demonstrated that all three fell within the normal range. Notably, in two of these cases (*AR* and *TBP*), sequencing read lengths were 100 bp, shorter than the minimum pathogenic repeat threshold. All three false positives originated from exome data. Importantly, no systematic increase in pathogenic-range calls was observed due to the reduced coverage default parameter of EH. Consistent with this, the distribution of EH-estimated repeat sizes (Figure 2A) showed no evidence of inflation in the pathogenic range, supporting the absence of a systematic increase in false-positive calls.

After excluding the two *AR* carrier cases and three false positives, PCR-based validation confirmed 23/28 EH-detected pathogenic alleles. Additionally, eight alleles initially estimated as intermediate by EH—four in *ATXN2*, three in *HTT*, and one in *DMPK*—were shown to be pathogenic by PCR. This increased the total number of pathogenic STR expansions identified in the study to 31, across nine loci in 28 families.

The distribution of pathogenic expansions among index cases included: *HTT* (*n* = 8), *ATXN2* (*n* = 5), *ATXN1* (*n* = 4), *DMPK* (*n* = 3), *PABPN1* (*n* = 3), *TBP* (*n* = 2), and one case each involving *AR*, *ATN1*, and *CACNA1A*. No pathogenic expansions were identified in the remaining loci of interest: *ATXN3*, *ATXN7*, *JPH3*, *PPP2R2B*, *THAP11*, and *ZFHX3*. The corresponding demographic, clinical, and molecular characteristics of each family, including prediagnosis, age at onset, family history, sequencing approach, EH repeat estimates, PCR-confirmed repeat sizes, and brief clinical descriptions of probands are summarized in Table 1. In light of the large number of findings across multiple disease loci, comprehensive clinical characterization, including longitudinal disease progression, neuroimaging, and detailed neurological assessment, will be presented in dedicated disease-specific follow-up studies.

After PCR-based reclassification of eight intermediate alleles as pathogenic, the total number of intermediate expansions decreased from 160 to 152. Figure 2C,D summarize the locus-specific and disease-category distribution of pathogenic and intermediate expansions following PCR refinement. Pathogenic expansions were most frequently detected in *HTT*, followed by *ATXN2* and *ATXN1*, together accounting for more than 60% of all pathogenic alleles. In contrast, intermediate-range expansions were predominantly observed in *ATXN2* and *ATXN1*, which collectively comprised over two-thirds of all intermediate alleles.

At the disease-category level, pathogenic expansions were most frequently identified in the ataxia (ATX) cohort, followed by ALS & FTD and myopathy (MYO) cases. Intermediate expansions were most prevalent in the ALS & FTD cohort, with lower frequencies observed in ATX and PD cases.

### 2.3. Comparison Between ExpansionHunter Repeat Estimation and PCR-Based Repeat Sizing

In addition to the eight intermediate alleles reclassified as pathogenic, we validated 69 out of 152 remaining intermediate-range alleles, focusing on those close to the pathogenic threshold, primarily in *ATXN2*, *ATXN1*, and *HTT* (Supplementary Tables S4 and S5). Intermediate alleles well below pathogenic borderline were not subjected to validation and were not considered for downstream analyses. Including previously identified cases with conventional methods (Supplementary Table S2), repeat size estimates generated by EH and PCR-based assays were available for 113 individuals across eight loci: *AR*, *ATN1*, *ATXN1*, *ATXN2*, *CACNA1A*, *HTT*, *PABPN1*, and *TBP*.

Pathogenic expansions in *DMPK* were assessed by fragment analysis to determine expansion status (pathogenic vs. non-pathogenic); however, precise repeat sizing was not available. Therefore, *DMPK* was excluded from the quantitative agreement analysis.

**Figure 3:**
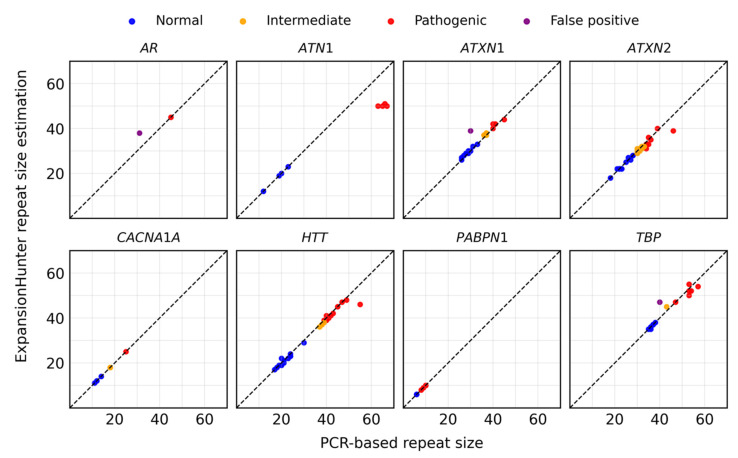
Agreement between ExpansionHunter repeat size estimates and PCR-based measurements. Comparison of repeat lengths estimated by EH and PCR-based validation in 113 individuals across eight loci: *AR*, *ATN1*, *ATXN1*, *ATXN2*, *CACNA1A*, *HTT*, *PABPN1*, and *TBP*. EH showed high concordance with PCR for both normal and expanded alleles, with only three false-positive calls.

Figure 3 illustrates the comparison between EH-derived repeat size estimates and PCR-based measurements. A per-gene summary of pathogenic, intermediate, normal, and false-positive calls is provided in Supplementary Table S6. Overall, EH demonstrated high agreement with PCR results, accurately estimating both normal (short) alleles and expanded alleles across loci. Only three false-positive calls were observed. Importantly, not all intermediate-range alleles were subjected to PCR validation. This is because intermediate expansions in the analyzed loci were shorter than the sequencing read-length and were therefore predominantly supported by spanning reads, for which EH demonstrated near-identical agreement with PCR measurements (typically within ±1–2 repeat units). Given this high accuracy in the size estimation, additional validation of all intermediate alleles was not considered necessary.

Five of the analyzed loci are known to harbor repeat motif interruptions (Supplementary Table S2). Despite this motif complexity, EH maintained robust performance across loci.

### 2.4. Motif Interruptions at ATXN1 Locus Analysis

Motif interruption analysis with REViewer tool was performed with particular focus on the *ATXN1* locus. Note that interruption motifs are reported based on the antisense (CTG) strand analyzed by ExpansionHunter/REViewer; thus, the detected (ATG) motif corresponds to (CAT) on the coding strand, which encodes histidine. While repeat interruptions may occur at other polyglutamine loci, interruptions at *ATXN1* alter the encoded amino acid sequence by introducing histidine residues within the polyglutamine tract.

The *ATXN1* locus was customized to genotype the (CTG)_n_ motif. Among the four EH-identified pathogenic *ATXN1* expansions, only one case (F04-P1), a clinically diagnosed ataxia patient, lacked histidine interruptions within the expanded allele (Supplementary Figure S8). The other two patients with two histidine interruptions as in the reference genome had clinical prediagnoses other than ataxia (Supplementary Figures S9 and S10).

Notably, the remaining patient, referred with a clinical prediagnosis of ALS, carried a 33/44 genotype with two additional histidine interruptions in the expanded allele (four in total). Based on REViewer alignments, the repeat configurations (5′ to 3′) of the short and expanded alleles were (Supplementary Figure S11):

- Short allele: Q_1–16_H_17_Q_18_H_19_Q_20–33_.
- Expanded allele: Q_1–14_H_15_Q_16_H_17_Q_18–29_H_30_Q_31_H_31_Q_33–44_.

All three individuals harboring pathogenic *ATXN1* expansions, despite differing initial clinical prediagnoses, were ultimately reclassified as spinocerebellar ataxia type 1 (SCA1) based on molecular findings and clinical re-evaluation.

Histidine (ATG) interruptions were detected via REViewer in all 15 PCR-confirmed intermediate cases (Supplementary Table S4).

### 2.5. Pathogenic HTT Repeat Expansions in Non-Huntington Disease Diagnoses

**Figure 4:**
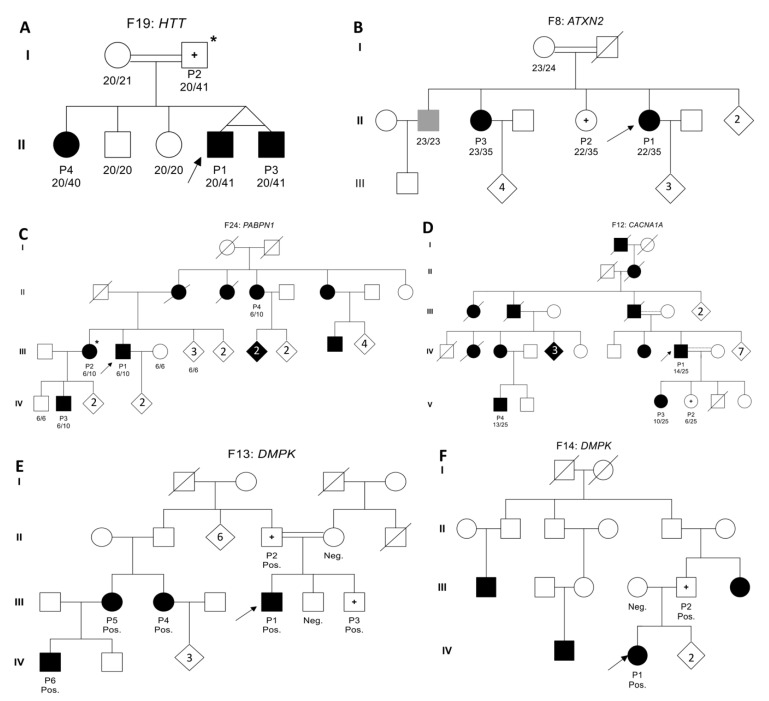
Pedigrees of families with segregating repeat expansions. (**A**) Family F19 with a pathogenic *HTT* expansion. (**B**) Family F8 with a pathogenic *ATXN2* expansion. (**C**) Family F24 with a pathogenic *PABPN1* expansion. (**D**) Family F12 with a pathogenic *CACNA1A* expansion. (**E**,**F**) Family F13 with a pathogenic *DMPK* expansion. Individuals marked with * have exome sequencing data in addition to the proband (indicated by an arrow). Squares represent males and circles represent females. Shaded symbols indicate affected individuals, while unshaded symbols indicate unaffected individuals. A plus sign (+) within a symbol indicates individuals who are positive for STR expansion but whose clinical status is unknown. Numbers within a symbol indicate additional siblings. Pos, positive; Neg, negative.

Eight *HTT*-positive cases with repeat lengths exceeding ≥40 (CAG)_n_ were referred with initial diagnoses other than HD. In a large family with five offspring, referred to us with a prediagnosis of ataxia, three siblings with congenital disease inherited the *HTT* expansion from a presymptomatic father. In contrast, the two unaffected siblings were shown to have normal repeat lengths (Figure 4A). Another patient with a juvenile-onset movement disorder at age five carried a 20/41 *HTT* expansion, also inherited from a presymptomatic father. One of the three remaining *HTT*-positive patients was initially diagnosed with PD and two with ALS. No additional segregating protein-altering mutations in relevant genes were identified in these families.

Additionally, six individuals with intermediate *HTT* alleles (36–39 repeats) were identified, who were clinically associated with ALS (*n* = 3), ataxia (*n* = 1), FTD (*n* = 1), and PD (*n* = 1) (Supplementary Table S4). These alleles fall within the reduced-penetrance range, and their clinical relevance in these cases remains uncertain.

### 2.6. ATXN2 Repeat Expansions

Given the established role of *ATXN2* intermediate expansions in ALS risk [6,11,14], all ALS patients and the majority of individuals with other clinical prediagnoses carrying ≥29 (CAG)_n_ repeats were validated via fragment analysis (Supplementary Tables S4 and S5). Based on the distribution of *ATXN2* repeat sizes across disease cohorts, intermediate-range alleles (29–33 repeats) were predominantly observed in ALS cohort (Supplementary Figure S12). Of the 53 intermediate alleles identified in the cohort, 34 (64.15%) were detected in ALS patients. In addition, three ALS individuals carried expansions at the borderline pathogenic threshold 34 repeats. In family F08, the 35-repeat allele segregated with ataxia in the proband and one sibling, while clinical data were unavailable for the other carrier (Figure 4B). The largest expansion (46 repeats) was detected in a patient with juvenile-onset ataxia, consistent with the established association between longer *ATXN2* expansions and spinocerebellar ataxia type 2 (SCA2). No intermediate or pathogenic expansions were observed in the healthy controls and myopathy cohorts.

### 2.7. Pathogenic Expansions in PABPN1 and Other Loci

To the best of our knowledge, this is the first report of genetically confirmed *PABPN1*-associated oculopharyngeal muscular dystrophy (OPMD) cases in Türkiye. Exome-based in silico STR genotyping revealed a heterozygous (GCG)_10_ expansion in two siblings with clinical OPMD; PCR confirmed segregation in four affected family members (Figure 4C). A heterozygous (GCG)_9_ was identified in an unrelated case with OPMD. Additionally, one neurologically healthy 61-year-old control individual carried a (GCG)_8_ allele, falling on the pathogenic borderline.

Pathogenic repeat expansions were identified in *TBP*, *ATN1*, *CACNA1A*, and *DMPK* with genotype–phenotype correlations consistent with established disease associations. The probands with *CACNA1A* and *DMPK* (F13) belonged to large sibships with multiple affected members. Fragment analysis confirmed that the expanded alleles co-segregated with disease (Figure 4D,E). Figure 4F depicts the pedigree of the *DMPK*-positive family, in which multiple individuals presented with similar symptoms; however, DNA samples were available only from the parents.

A single pathogenic expansion in *AR* was detected in a patient initially diagnosed with motor neuron disease. Following molecular confirmation and subsequent clinical re-evaluation, the diagnosis was revised to spinal and bulbar muscular atrophy.

### 2.8. Diagnostic Yield

Using in silico genotyping with EH on short-read NGS data, we uncovered the genetic basis in 27 previously unsolved families; three of these families had additional affected family members with available sequencing data. In total, 30 individuals within the patient cohort with NGS data received a molecular diagnosis through this approach. The molecular diagnoses were largely consistent with the clinical prediagnoses. This corresponds to an additional diagnostic yield of ~0.95% (30/3150 patients). Notably, a neurologically normal control individual harboring a minimally pathogenic expansion in the *PABPN1* locus was excluded from this calculation.

## 3. Discussion

Transformative bioinformatic advances in NGS-based STR genotyping have enabled the systematic screening of large disease cohorts for the detection of clinically relevant repeat expansions, particularly in neurological disorders [3,8,11,12,13,14,15,16]. Such studies help to resolve genetic heterogeneity and improve differential diagnosis. NGS-based STR genotyping has the potential to complement or replace site-specific conventional methods for many loci.

The present cohort was recruited between 2005 and 2025 at NDAL, a nationwide reference center for the genetics of neurodegenerative diseases in Türkiye. All samples had previously undergone comprehensive analysis for protein-altering variants, short indels, and copy number variants. Targeted repeat expansion findings were obtained through conventional methods in specific disease groups (e.g., (CAG)_n_ triplet-repeat for hereditary ataxias), for which short-read exome/genome sequencing were considered uninformative [10,17,18,19]. However, most patients had not been systematically screened for repeat expansions across the 15 STR loci investigated in this study.

By integrating systematic in silico STR genotyping into the existing genomic workflow, we achieved an additional diagnostic yield of 0.95%, thereby unraveling 27 previously unsolved cases. In addition, we identified 160 intermediate-range alleles across multiple loci, of which 77 were validated by orthogonal methods. Given the elevated mutation rates of STRs and their tendency for further expansion across generations (genetic anticipation), intermediate alleles are clinically important, particularly in the context of incomplete penetrance observed at certain loci, such as *HTT* [1]. Collectively, these findings underscore the incremental diagnostic and prognostic value of incorporating targeted repeat expansion screening into routine genomic pipelines, particularly in clinically heterogeneous neurogenetic cohorts.

In this study, we identified a pathogenic repeat expansion in the *AR* locus in a male patient initially diagnosed with motor neuron disease. Following molecular confirmation and clinical re-evaluation, the diagnosis was revised to spinal and bulbar muscular atrophy, consistent with similar observations reported in Norwegian ALS cohorts [14].

### 3.1. Interruptions in ATXN1 Modulate SCA1 Penetrance and Clinical Manifestation

The presence of (ATG)_n_ interruptions in the *ATXN1* repeat locus plays an important role in modulating the SCA1 phenotype [20,21]. Among the four pathogenic *ATXN1* carriers in our cohort, only the patient without interruptions in the expanded allele presented with ataxia symptoms. The other three patients, whose expanded alleles contained (ATG)_n_ interruptions, were referred with clinical diagnoses of ALS or PD. Notably, Frontali et al. described a healthy 66-year-old father and his 24-year-old son who both carried a 45-repeat *ATXN1* allele containing four histidine interruptions [22]. In contrast, our index patient developed initial symptoms of dysphagia and dysarthria at the age of 66, despite harboring an expanded allele with a comparable total of four histidine interruptions within the polyglutamine tract. This observation is consistent with previous reports suggesting that histidine interruptions in *ATXN1* may influence phenotypic variability; the small number of cases in our cohort limits our ability to perform a quantitative assessment of interruption patterns and their correlation with age at onset, disease severity, or progression rate.

### 3.2. Infrequent Atypical Clinical Profiles of HTT Expansions

Intermediate *HTT* alleles (36–39 repeats) are generally associated with reduced or age-dependent penetrance, whereas alleles ≥40 repeats are considered pathogenic [1], although they may present with atypical clinical features beyond classical Huntington’s disease. Atypical *HTT*-related presentations have been reported previously. Squitieri et al. found Parkinsonism, dystonia, or ataxia at onset in 15 of 205 HD patients [9]. In line with this, Dong et al. identified seven probands initially diagnosed with spinocerebellar ataxia, of whom only three later developed chorea [23]. Méreaux et al. detected only two intermediate *HTT* alleles in 498 exomes from patients with spinocerebellar ataxia, suggesting incidental findings [13].

In accordance with previous studies, we identified five *HTT*-positive patients initially diagnosed with ataxia (40–55 repeats); notably, two inherited the expansion from an asymptomatic father and presented with congenital ataxia despite repeat sizes near the minimum pathogenic threshold. We also detected four intermediate and two pathogenic *HTT* expansions among ALS patients, replicating prior observations and underscoring the rarity of *HTT* expansions in the genetic architecture of ALS and FTD [8,11,14]. Collectively, these findings highlight the clinical heterogeneity of *HTT* expansions and are consistent with previously reported atypical presentations. However, alternative explanations such as incidental findings or phenocopies cannot be excluded, particularly in cases with repeat sizes near the pathogenic threshold or in the presence of overlapping neurodegenerative phenotypes.

### 3.3. First Genetically Confirmed OPMD Cases from Türkiye

Bilgen et al. reported a Turkish male patient with a family history of OPMD without genetic confirmation [24]. This early study was followed by the identification of a biallelic (GCN)_13_ expansion in the *PABPN1* gene in a female patient of Turkish origin within a large OPMD cohort using conventional genotyping [25]. We identified for the first time two unrelated OPMD cases carrying pathogenic *PABPN1* repeat expansions from Türkiye. In addition, we detected (GCG)_8_ expansion, the pathogenicity borderline for *PABPN1*, in a neurologically healthy 61-year-old control individual of Turkish-Persian origin, born to healthy parents. The smallest pathogenic *PABPN1* expansion, (GCG)_8_, is generally associated with a milder disease course, with symptoms, typically limited to ptosis and mild dysphagia, appearing later in life, often in the seventh decade [26]. This reduced penetrance leading to attenuated phenotype may explain the absence of OPMD symptoms in our control individual.

### 3.4. Recently Identified Triplet Expansions in THAP11 and ZFHX3 in Ataxia Phenotype

Repeat expansions in *THAP11* (SCA51) and *ZFHX3* (SCA4) were recently described in Chinese and Swedish families, respectively. However, follow-up studies suggest that they are extremely rare causes of ataxia [27,28,29,30]. Consistent with this, we found no pathogenic alleles at either locus in our large Turkish cohort, including more than 500 ataxia cases. These results, along with prior reports, suggest that *THAP11* and *ZFHX3* expansions are likely to represent geographically restricted or rare causes of ataxia. The ability to incorporate recently identified loci into EH-based analysis pipelines highlights the flexibility of NGS-based STR genotyping, enabling rapid retrospective screening of large cohorts with high accuracy while minimizing the need for additional laboratory testing.

### 3.5. Technical Considerations

Sequencing read length and STR locus selection are critical determinants of genotyping accuracy using EH. Repeat lengths shorter than the sequencing read length are typically estimated with high accuracy [3,10,15]. Minor discrepancies of ±1–2 repeat units between EH spanning-read calls and PCR measurements are generally considered acceptable, reflecting inherent sizing limitations of conventional methods (Figure 3) [13,31]. In our cohort, alleles supported by spanning reads showed strong concordance with PCR results, usually differing by only ±1–2 repeat units. In contrast, when repeat lengths exceeded the read length, size estimation relied primarily on flanking or in-repeat reads. Under these conditions, EH tended to underestimate expansion size, as observed in pathogenic *ATN1* alleles. Correspondingly, genotype confidence intervals were broader, indicating the possibility of larger repeat sizes. Notably, 93.27% of samples were sequenced with 150 bp reads, which were sufficient to accurately genotype all target loci up to the minimum pathogenic threshold. Only three false-positive calls were observed, two of which occurred in samples with 100 bp reads. Studies evaluating larger STR panels have reported higher false-positive rates [32,33]. Collectively, these findings indicate that the selected STR loci can be reliably genotyped in exome and genome datasets, particularly when adequate read length is available.

Locus coverage is a critical determinant of STR genotyping performance in exome sequencing due to variability introduced by different exome capture platforms. As shown in Supplementary Figure S2, coverage across target STR loci differed markedly between capture kits. Similar challenges have been reported previously, with key loci such as *ATXN2* often insufficiently captured (e.g., 8.8% genotyping with TruSeq; exclusion with SureSelect V5/V6) [15,16]. These findings highlight that STR genotyping efficiency using EH in exome data is highly dependent on local read depth. To address this limitation, we lowered the default locus coverage threshold from 10× to 3×. This adjustment substantially improved genotyping performance for poorly captured loci, increasing the genotyping rate for *ATXN2* to 89.6%, representing an approximately 10-fold improvement compared to previous reports. Strikingly, all pathogenic and majority of intermediate *ATXN2* expansions identified in our exome dataset had coverage values below 10×, indicating that the default threshold would have failed to detect them. Overall, genotyping rates across all target loci exceeded 70%, with some loci achieving complete (100%) genotyping in exome samples (Supplementary Figure S13). Importantly, the combination of a relaxed coverage threshold and visual inspection using REViewer enabled recovery of true expansions in poorly captured exons without introducing additional false positives.

## 4. Materials and Methods

### 4.1. Study Cohort

The study cohort includes 3150 cases and 146 healthy controls collected over 20 years across Türkiye, including a small number of cases of Azerbaijan origin, all evaluated at the Neurodegeneration Research Laboratory (NDAL). Genomic DNA was extracted from whole blood using the Qiagen EZ1 Advanced XL system (QIAGEN, Hilden, Germany). Exome sequencing (ES) was conducted on individuals diagnosed with neurodegenerative, neurological, or ultra-rare genetic disorders (2240 cases). Genome sequencing (GS) was carried out as part of Project MinE consortium on 910 sporadic ALS and ALS/FTD patients, along with 146 neurologically healthy controls (https://projectmine.com/, accessed on 4 March 2026). Control samples were included to assess the presence of pathogenic repeat expansions but were not used for comparative frequency analyses. Their demographic characteristics are provided in Supplementary Table S7. Typically, NGS was performed on affected probands who were clinically diagnosed by expert clinicians on neuromuscular and/or movement disorders. EH was applied to the entire cohort without excluding the samples with previously identified gene variants.

### 4.2. Alignment of Short-Read Sequencing Data

Genome sequencing data were processed within the framework of Project MinE, aligned to GRCh38, and prepared using the project’s standardized pipeline as described in van Rheenen, W. et al. [34].

The majority of exome samples were sequenced (Macrogen, Seoul, Republic of Korea) using Agilent SureSelect Human All Exon V6 kit (Agilent Technologies, Santa Clara, CA, USA) with 150 bp paired-end reads (Supplementary Table S1). Raw FASTQ files were quality-checked using FastQC v0.11.9 to assess per-cycle quality, adapter content, and GC bias. Trimming of adapters and low-quality bases was performed with fastp v0.23.4 using default parameters [35,36].

The cleaned reads were aligned to the GRCh38 human reference genome using BWA-MEM v0.7.17 with the -M option and piped directly to samtools v1.14 for sorting and indexing. Mate-pair information was corrected using GenomeAnalysisToolkit v4.6.1 (GATK) FixMateInformation (v4.6.1), and polymerase chain reaction (PCR)/optical duplicates were flagged using GATK MarkDuplicates [37,38,39].

Base quality scores were recalibrated using GATK BaseRecalibrator and ApplyBQSR, producing final alignment files that were duplicate-marked, recalibrated, and coordinate-sorted. These served as input for downstream analyses.

### 4.3. In Silico Genotyping of Short Tandem Repeat Loci

Computational STR genotyping was performed using EH v5 on the aligned files [10]. The variant catalog was customized to target codon-matching repeat motifs, and comprising motif interruptions, such as in the *HTT* locus.

Given the variable coverage of exome across sequencing kits compared with genome (Supplementary Figures S1 and S2), the minimum required locus coverage was reduced from the default of 10× to 3× to improve the genotyping rate [15,16]. Fifteen disease-associated STR loci—*AR*, *ATN1*, *ATXN1*, *ATXN2*, *ATXN3*, *ATXN7*, *CACNA1A*, *DMPK*, *HTT*, *JPH3*, *PABPN1*, *PPP2R2B*, *TBP*, *THAP11*, *ZFHX3*—were analyzed. Since patients with ALS and FTD were routinely genotyped for the pathogenic *C9orf72* repeat expansion prior to NGS, this locus was not included in the present study [18]. Accordingly, patients with alleles exceeding the established pathogenic threshold (>30 repeat units) [1] were not sequenced for either exome or genome. Furthermore, as the *C9orf72* repeat is located within a non-coding intronic region that is not reliably captured by most exome sequencing kits [1,10], its assessment from exome data would not be technically appropriate. The distribution of *C9orf72* repeat sizes in our genome dataset is presented separately in Supplementary Figure S3.

### 4.4. Validation of Potential Repeat Expansions

PCR-based validation was performed using Sanger sequencing or fragment analysis (Macrogen, Seoul, Republic of Korea), depending on the locus characteristics for all pathogenic repeats, except for *AR* carriers and some of intermediate expansions (Supplementary Table S2).

Sanger results were visualized and analyzed using FinchTV v1.4.0 (Geospiza Inc., Seattle, WA, USA), and fragment analysis results were analyzed with Peak Scanner Software v1.0 (Thermo Fisher Scientific, Waltham, MA, USA).

### 4.5. Data Analysis and Visualization

Repeat sizes inferred by EH were classified as normal, intermediate, or pathogenic according to the thresholds listed in Supplementary Table S2. Data analyses and visualizations were conducted in Python 3. Finally, clinically relevant expanded alleles identified by EH were visually inspected using REViewer v0.2.7 to observe alignment patterns and motif interruptions [40]. As REViewer is specifically designed to visualize ExpansionHunter output, EH was used to genotype STRs at loci of interest in this study.

ExpansionHunter and REViewer operate on the reference genome in the 5′ to 3′ direction. Accordingly, repeat motifs may be represented on the antisense strand; for example, the canonical (CAG) repeat on the coding strand corresponds to (CTG) on the antisense strand. Consequently, the interruption motif (ATG) detected by EH in the *ATXN1* locus corresponds to (CAT) on the coding strand, which encodes histidine.

## 5. Conclusions

NGS-based in silico STR genotyping with EH enables systematic detection of disease-associated expansions, providing new molecular diagnoses and revealing atypical clinical presentations. Our findings highlight the genetic heterogeneity of neurodegenerative diseases, especially movement disorders, and the importance of careful visual evaluation of the repeat structure. This study enhances diagnostic accuracy in complex neurodegenerative disorders and expands future neurogenetic research.

## Figures and Tables

**Table 1:** Clinical features and genotyping results for individuals carrying pathogenic expansions at the targeted loci.

Family ID	Individual ID	Clinical Prediagnosis	Gender	Date of Birth	Age on Onset	Gene	NGS Type	EH Genotype	EH Repeat Range	Repeat Size (PCR-Based)	Clinical Presentation of Probands
F01	F01-P1	ALS	M	1943	70	*AR*	GS	45	45–54	45	Tongue atrophy, fasciculations, paraplegia.
F02	F02-P1	ataxia	M	1973	38	*ATN1*	ES	12/51	12–12/50–55	12/66	Gait ataxia, dysarthria, sensory ataxia (Romberg+), deep sensory involvement, cognitive impairment, cerebral-cerebellar atrophy.
F03	F03-P1	ALS	F	1958	66	*ATXN1*	ES	33/44	33–33/44–47	33/45	Dysphagia, Dysarthria
F04	F04-P1	ataxia	F	1960	50	*ATXN1*	ES	30/42	30–30/42–42	30/40	Gait ataxia and gait imbalance.
F05	F05-P1	PD	M	1977	43	*ATXN1*	ES	29/40	29–29/40–40	29/40	Tremor, bradykinesia on the right side
F05	F05-P2		M	1942		*ATXN1*				29/40	
F06	F06-P1	ALS	F	1955	58	*ATXN1*	GS	29/42	29–29/42–42	28/41	Unilateral weakness in extremities
F07	F07-P1	ataxia	F	1999	16	*ATXN2*	ES	22/39	22–22/38–62	23/46	Gait ataxia, dysarthria, dysmetria, polyneuropathy (sensory and axonal), deep sensory involvement, cerebellar-spinal atrophy.
F08	F08-P1	ataxia	F	1975	20	*ATXN2*	ES	22/33	22–22/32–45	22/35	Complex phenotype, weakness in legs, difficulty in climbing stairs, swallowing difficulty
F08	F08-P2		F			*ATXN2*				22/35	
F08	F08-P3		F	1970	22	*ATXN2*				23/35	
F09	F09-P1	ALS	M	1975	45	*ATXN2*	ES	22/33	22–22/33–49	22/34	Progressive disturbances in speech
F10	F10-P1	ALS	F	1958	63	*ATXN2*	ES	22/31	22–22/31–32	22/34	Difficulty in walking
F11	F11-P1	ALS	M	1959	58	*ATXN2*	GS	22/33	22–22/33–33	22/34	Weakness in arms, cramps in the legs
F12	F12-P1	ataxia	M	1968	32	*CACNA1A*	ES	14/25	14–14/25–25	14/25	Progressive ataxia, ataxic gait, cerebellar dysarthria, cerebellar atrophy
F12	F12-P2		F	1989		*CACNA1A*				6/25	
F12	F12-P3		F	1986	34	*CACNA1A*				10/25	
F12	F12-P4		M	1991	29	*CACNA1A*				13/25	
F13	F13-P1	myopathy	M	1985	34	*DMPK*	ES	48/48	37–65/47–79	positive	Progressive muscle weakness
F13	F13-P2		M			*DMPK*				positive	
F13	F13-P3		M	1992		*DMPK*				positive	
F13	F13-P4		F			*DMPK*				positive	
F13	F13-P5		F			*DMPK*				positive	
F13	F13-P6		M			*DMPK*				positive	
F14	F14-P1	myopathy	F	2001	adolescent	*DMPK*	ES	32/53	32–32/49–66	positive	Myotonia
F14	F14-P2		M			*DMPK*					
F15	F15-P1	myopathy	F	1963	53	*DMPK*	ES	11/55	11–11/50–97	positive	Proximal weakness, imbalance, drooping of one eyelid
F16	F16-P1	ataxia	M	1971	40	*HTT*	ES	29/48	29–29/48–49	30/49	Gait ataxia, dysarthria, gait imbalance
F17	F17-P1	ataxia	F	1995	23	*HTT*	ES	23/46	23–23/46–52	24/55	Gait ataxia, upper motor neuron involvement, gait imbalance.
F18	F18-P1	ataxia	F	1969	49	*HTT*	ES	23/45	23–23/44–49	24/45	Gait ataxia, gait imbalance, upper motor neuron involvement, lower extremity involvement, chorea
F19	F19-P1	ataxia	M	2010	congenital	*HTT*	ES	19/40	19–19/40–44	20/41	Very low birth weight (1200 g). Gait imbalance began at age 2, with cerebellar and pyramidal signs on examination.
F19	F19-P2		M	1979		*HTT*	ES	19/40	19–19/40–40	20/41	
F19	F19-P3		M	2010	congenital	*HTT*				20/41	
F19	F19-P4		F	1996	congenital	*HTT*				19/40	
F20	F20-P1	ataxia	M	1989	5	*HTT*	ES	19/40	19–19/40–40	20/41	Cerebellar ataxia, pyramidal signs.
F20	F20-P2		M	1964		*HTT*	ES			20/40	NA
F21	F21-P1	PD	M	1969	50	*HTT*	ES	22/39	22–22/39–39	23/40	NA
F22	F22-P1	ALS	F	1943	69	*HTT*	GS	19/41	19–19/41–41	20/42	Typical ALS
F23	F23-P1	ALS	F	1946	70	*HTT*	GS	20/39	20–20/39–39	21/40	Unilateral weakness, tongue atrophy
F24	F24-P1	OPMD	M	1959	55	*PABPN1*	ES	6/10	6–6/10–10	6/10	Progressive ptosis, dysphagia and dysarthria over 10 years
F24	F24-P2		F	1956	55	*PABPN1*	ES	6/10	6–6/10–10	6/10	
F24	F24-P3		M	1981		*PABPN1*				6/10	
F24	F24-P4		F			*PABPN1*				6/10	
F25	F25-P1	OPMD	F	1954	33	*PABPN1*	ES	6/9	6–6/9–9	6/9	Bilateral ptosis, progressive proximal muscle weakness, difficulty swallowing
F26	F26-C1	control	M	1954		*PABPN1*	GS	6/8	6–6/8–8	6/8	Neurologically healthy
F27	F27-P1	ataxia	M	1985	27	*TBP*	ES	37/54	37–37/52–59	37/57	Gait ataxia, dysarthria, gait imbalance, nystagmus, dysmetria, dysdiadochokinesis, epilepsy, cerebral-cerebellar atrophy, sleep disturbance.
F28	F28-P1	ataxia	F	2003	9	*TBP*	ES	36/47	36–36/47–47	36/47	Gait ataxia, dysarthria, intellectual disability, sepsis hyperbilirubinemia

ALS: amyotrophic lateral sclerosis; EH: ExpansionHunter; OPMD: oculopharyngeal muscular dystrophy; PD: Parkinson’s disease; ES: Exome Sequencing; GS: Genome sequencing. NA: Not Available.

## Data Availability

The data that support the findings of this study are available from the corresponding author upon request.

## Supplementary Materials

The following supporting information can be downloaded at: https://www.mdpi.com/article/10.3390/ijms27104345/s1.

## Abbreviations

The following abbreviations are used in this manuscript:

ALS	Amyotrophic Lateral Sclerosis
ATX	Ataxia
CNV	Copy Number Variation
EH	ExpansionHunter
ES	Exome Sequencing
FTD	Frontotemporal Dementia
GATK	GenomeAnalysisToolkit
GS	Genome Sequencing
HD	Huntington disease
MYO	Myopathy
NDAL	Neurodegeneration Research Laboratory
NGS	Next Generation Sequencing
OPMD	Oculopharyngeal muscular dystrophy
PCR	Polymerase Chain Reaction
PD	Parkinson’s Disease
SCA	Spinocerebellar ataxia
STR	Short Tandem Repeat
