# Supplementary material for: Computational Short Tandem Repeat Genotyping Reveals Clinically Relevant Expansions in a Large Turkish Neurodegeneration Disease Cohort
Source: Int J Mol Sci. 2026 May 13;27(10):4345. doi: 10.3390/ijms27104345 (PMC13207311; doi:10.3390/ijms27104345)
Supplement: Supplementary file 1 [file ijms-27-04345-s001.zip › supp.pdf]

Supplementary Materials for

Computational Short Tandem Repeat Genotyping Reveals Clinically Relevant Expansions in a Large Turkish Neurodegeneration Disease Cohort

Khojakulov Z. et al.

This Supplementary file includes:

- Figures S1 to S13
- Tables S1 to S7

Table S1. Short-read NGS kits used in the study.

NGS type	kit	Read Length	Sample Count
**GS**	Hiseq X	150	255
	NovaSeq6000	150	801
	**Total**		**1056**
**ES**	Roche SeCap EZ Whole Exome V2	100	7
	Roche SeqCap EZ Whole Exome V3	100	27
	Agilent SureSelect Human All Exon V5	100	105
	Agilent SureSelect Human All Exon V5	125	21
	Agilent SureSelect Human All Exon V6	100	126
	Agilent SureSelect Human All Exon V6	150	1882
	TruSeq Exome Library Prep Kit	110	64
	Xgen exome research panel V2	100	8
	**Total**		**2240**

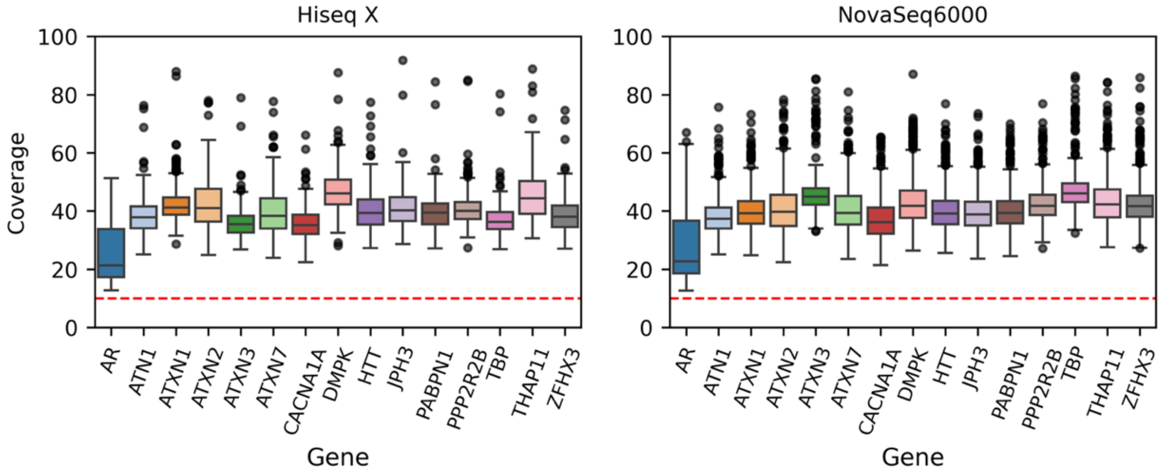

Figure S1. Distribution of locus-level read coverage across 15 STR loci in genome data stratified by library preparation kit. Each panel represents one genome kit (150-bp read length). Dashed-red line represent 10× reads required to call ExpansionHunter with default settings.

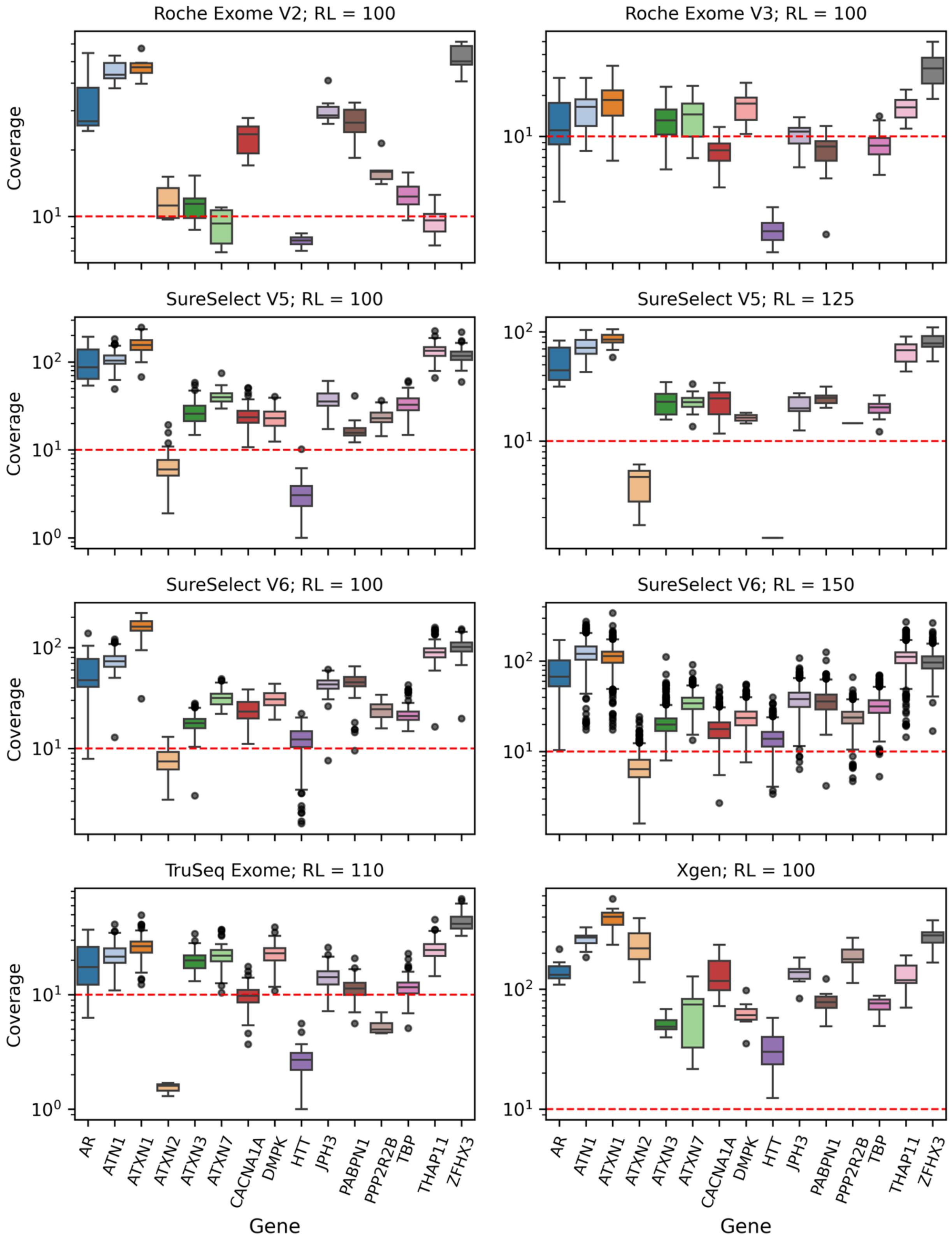

Figure S2. Distribution of locus-level read coverage across 15 STR loci in exome data stratified by library preparation kit and read-length. Coverage across loci showed substantial variability

between kits and genomic regions. The Roche V3 kit (100-bp reads) showed lack of coverage for the *ATXN2* and *PPP2R2B* loci. Dashed-red line represent 10× reads required to call ExpansionHunter with default settings. The y-axis is presented on a logarithmic scale. **Note.** The number of samples for each kit is provided in **Table S1**. The majority of samples (1882/2240; 84%) were generated using the Agilent SureSelect V6 kit with 150-bp paired-end reads.

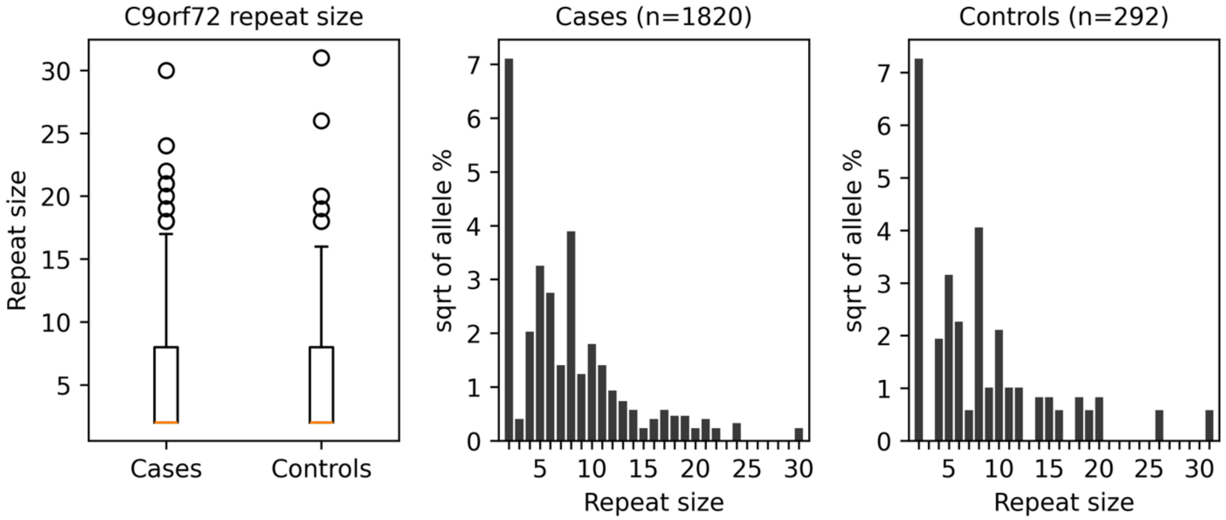

Figure S3. Distribution of *C9orf72* repeat sizes in 910 sporadic ALS cases and 146 controls from the genome sequencing dataset. All ALS cases were previously screened for pathogenic *C9orf72* expansions using standard clinical assays; therefore, no long expansion-positive individuals were present in this cohort. The observed distribution reflects non-expanded alleles and demonstrates concordance between prior clinical testing and in silico genotyping. Repeat sizes of 2, followed by 8 were the most frequent alleles both in cases and controls groups, together accounting for more than half of all observed alleles. *n* denotes the total number of alleles analyzed.

Table S2. Repeat loci in 15 genes. Diseases associated, gene region, repeat motif with interruption information, amino acid, thresholds, and validation methods are given. Motif interruption at *ATXN1* and *ZFHX3* loci changes into histidine and serine, respectively.

Gene	Disease	MoI	Location (GRCh38/hg38)	Gene region	Motif	Encoding amino acid	Motif inter.	Normal upper bound	Intermediate range	Pathogenic lower bound	Validation
AR	Spinal and bulbar muscular atrophy	XLR	chrX:67545317-67545383	coding	CAG	Q	pure	34	35-37	38	Standard PCR
ATN1	Dentatorubral-pallidoluysian atrophy	AD	chr12:6936716-6936773	coding	CAG	Q	CAA	35	36-47	48	Standard PCR
ATXN1	Spinocerebellar ataxia 1	AD	chr6:16327635-16327722	coding	CTG	Q	ATG	35	36-38	39	Fragment analysis
ATXN2	Spinocerebellar ataxia 2	AD	chr12:111598950-111599019	coding	CTG	Q	TTG	28	29-33	34	Fragment analysis
ATXN3	Spinocerebellar ataxia 3	AD	chr14:92071010-92071040	coding	CTG	Q	TTG	44	45-54	55	
ATXN7	Spinocerebellar ataxia 7	AD	chr3:63912685-63912715	coding	CAG	Q	pure	33	34-36	37	
CACNA1A	Spinocerebellar ataxia 6	AD	chr19:13207858-13207897	coding	CTG	Q	pure	17	18-19	20	Fragment analysis
DMPK	Myotonic dystrophy 1	AD	chr19:45770204-45770264	UTR	CAG		pure	37	38-50	51	Fragment analysis
HTT	Huntington disease	AD	chr4:3074876-3074939	coding	CAG	Q	CAA	35	36-39	40	Fragment analysis
JPH3	Huntington disease-like 2	AD	chr16:87604287-87604329	UTR	CTG		pure	28	29-40	41	
PABPN1	Oculopharyngeal muscular dystrophy	AD	chr14:23321472-23321490	coding	GCG	A	pure	6	7	8	Standard PCR
PPP2R2B	Spinocerebellar ataxia 12	AD	chr5:146878728-146878758	UTR	CTG		pure	32	33-42	43	
TBP	Spinocerebellar ataxia 17	AD	chr6:170561907-170562021	coding	CAG	Q	CAA	42	43-46	47	Fragment analysis
THAP11	Spinocerebellar ataxia 51	AD	chr16:67842863-67842950	coding	CAG	Q	CAA	38	39-46	47	
ZFHX3	Spinocerebellar ataxia 4	AD	chr16:72787694-72787757	coding	GCC	G	ACC/ACT	31	32-41	42	

Notes: Disease-associated STR loci summarized in the review by Depienne [1]. MoI - Mode of Inheritance, inter - interruption, ref – reference, UTR- untranslated region.

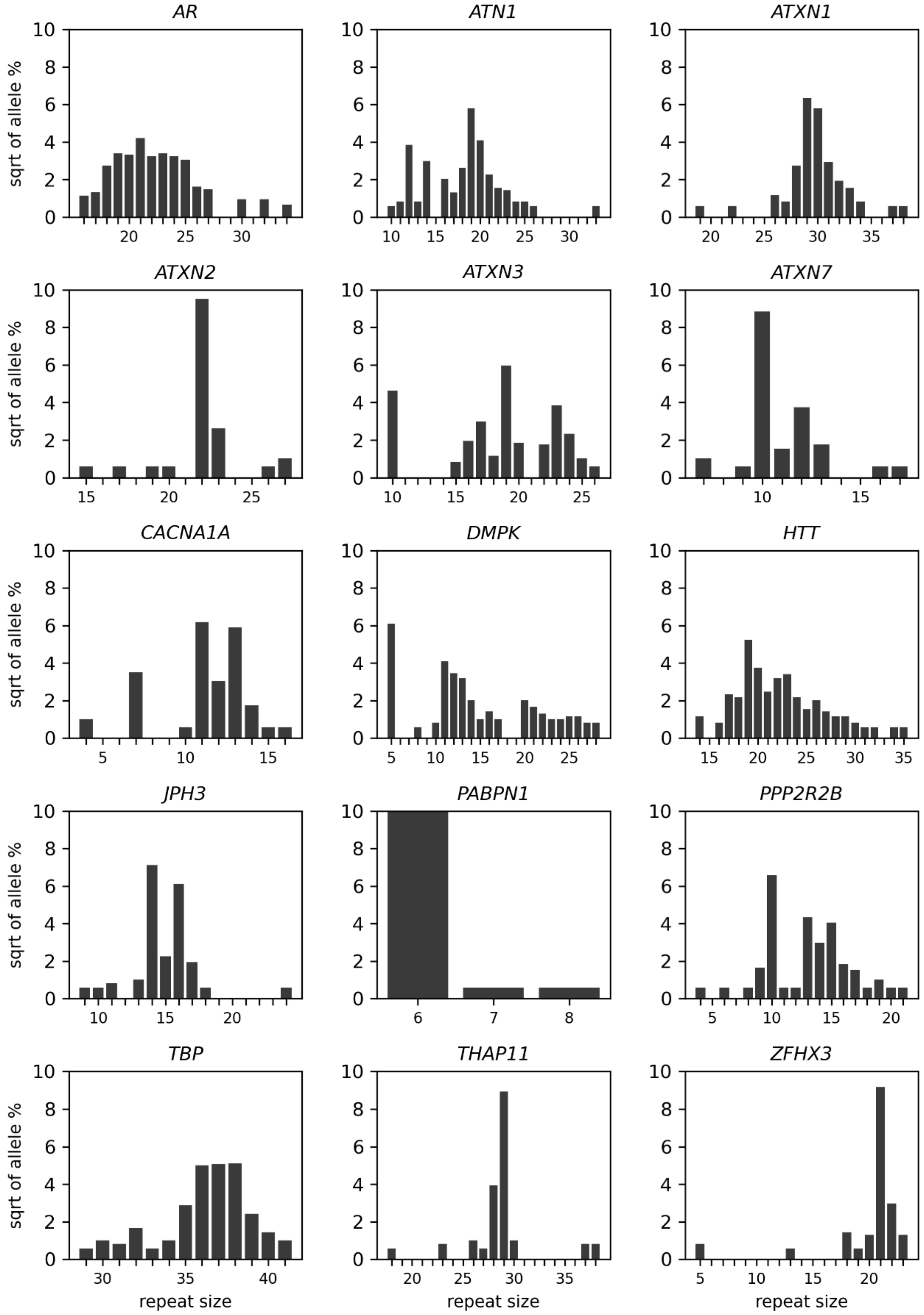

Figure S4. Percentage distribution of repeat sizes across 15 loci in 146 control individuals. The total allele count was 226 at the *AR* locus and 292 at each of the remaining loci. The reduced allele number at *AR* reflects its X-linked inheritance.

Table S3. Comparison between ExpansionHunter genotyping and prior wet-laboratory results in previously diagnosed cases. The genotype interval of ExpansionHunter should be considered when the repeat length is close to or exceeds the sequencing read length.

Family ID	Individual ID	Clinical prediagnosis	Gender	Date of Birth	Age on onset	Gene	ExpansionHunter		PCR-based Repeat Size
							Genotype	Genotype interval	
V01	V01-P1	ataxia	F	1958	39	*ATN1*	19/50	19-19/50-63	19/63
V01	V01-P2	ataxia	M	1974	30	*ATN1*	23/50	23-23/50-64	23/67
V01	V01-P3	ataxia	F	1973	35	*ATN1*	20/50	20-20/50-58	20/65
V02	V02-P1	ataxia	F	1970	44	*ATXN2*	22/40	22-22/40-40	22/39
V03	V03-P1	ataxia	M	1999	14	*ATXN2*	22/35	22-22/35-52	21/36
V03	V03-P2	ataxia	M	1959	NA	*ATXN2*	22/36	22-22/36-36	22/35
V04	V04-P1	HD	F	1972	48	*HTT*	17/47	17-17/47-47	17 / 47
V05	V05-P1	HD	F	1956	46	*HTT*	24/42	24-24/42-42	24/43
V06	V06-P1	HD	F	1946	65	*HTT*	18/41	18-18/41-41	18/40
V07	V07-P1	HD	F	1956	60	*HTT*	19/39	19-19/39-39	19/39
V08	V08-P1	ataxia	F	1976	36	*TBP*	38/55	38-38/51-63	38/53
V08	V08-P2	ataxia	F	NA	50	*TBP*	37/52	37-37/50-60	37/53
V09	V09-P1	ataxia	F	1977	17	*TBP*	37/52	37-37/52-59	37/54
V10	V10-P1	ataxia	F	1961	35	*TBP*	35/50	35-35/50-55	35/53

Note: All these patients have exome data.

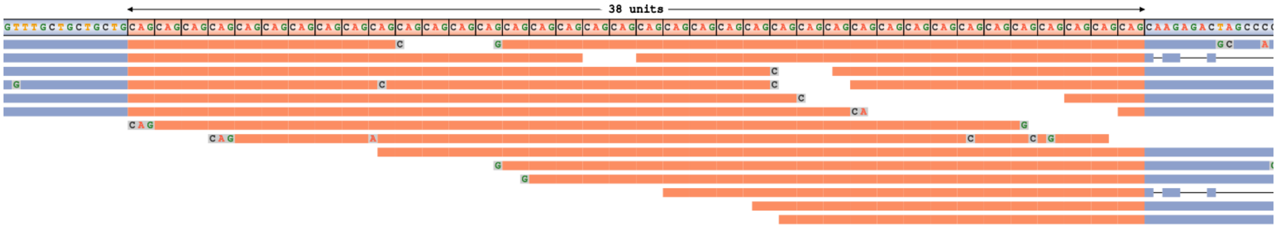

Figure S5. REViewer visualization of the *AR* repeat locus (male). ExpansionHunter estimated 38 repeats (genotype interval: 33–52), with a locus coverage of 38.7×. The 100 bp read length of the exome data likely limited accurate sizing of the repeat, as PCR validation confirmed 31 repeats.

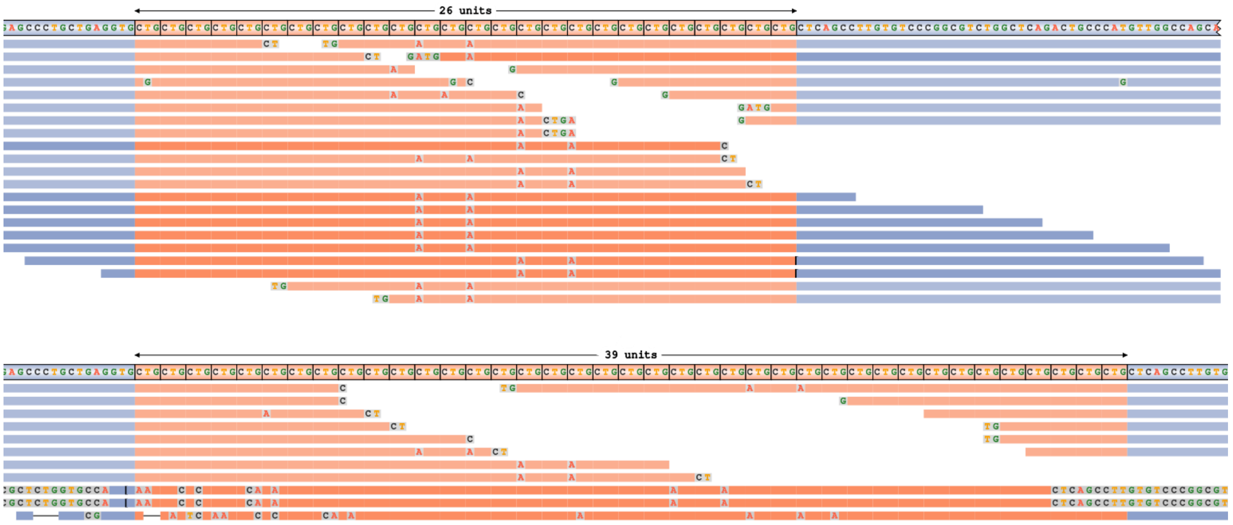

Figure S6. REViewer visualization of the *ATXN1* repeat locus. ExpansionHunter estimated 26/39 repeats (genotype interval: 26-26/35-39), with a locus coverage of 107,3×. The gaps in alignments in expanded alleles are likely limited accurate sizing of the repeat, as PCR validation confirmed 26/30 repeats.

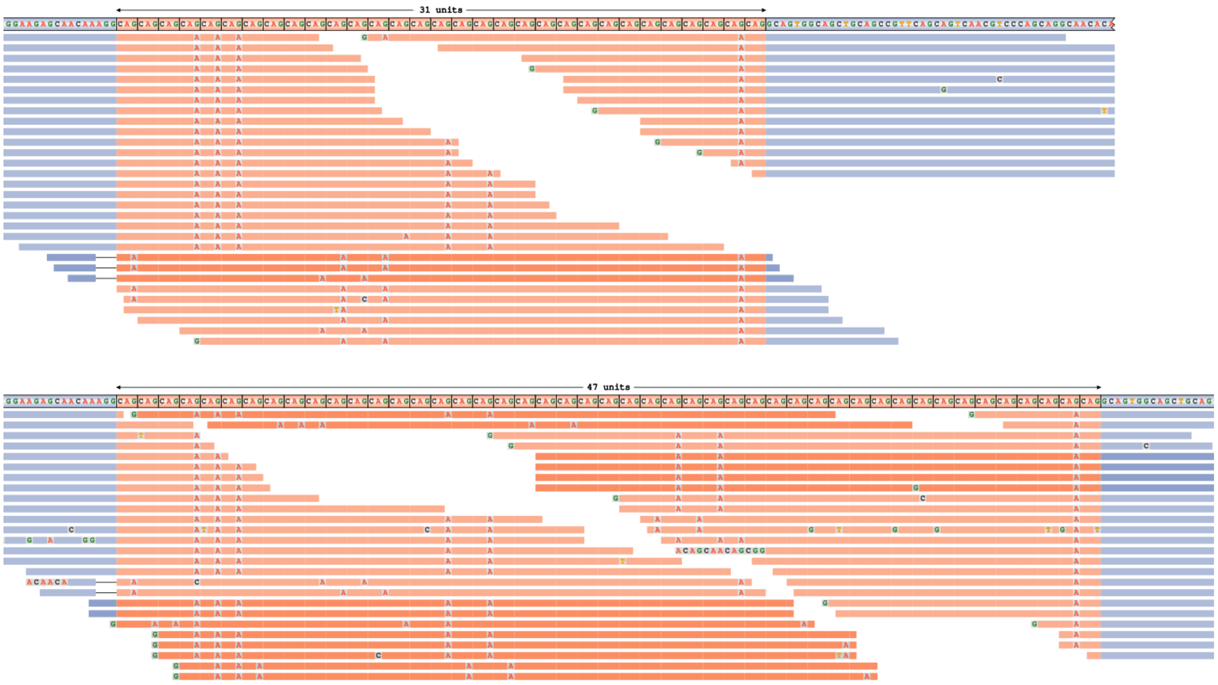

Figure S7. REViewer visualization of the TBP repeat locus. ExpansionHunter estimated 31/47 repeats (genotype interval: 31-42/39-55), with a locus coverage of 19.6×. The 100 bp read length of the exome data likely limited accurate sizing of the repeat, as PCR validation confirmed 36/40 repeats.

Table S4. Thirty-five individuals harboring intermediate range expansions identified by ExpansionHunter and confirmed with conventional methods.

ID	Clinical prediagnosis	Gender	Date of Birth	Age on Onset	NGS Type	Gene	EH genotype	PCR-based
I1	FTD	F	1972	42	ES	*ATXN1*	29/38	29/37
I2	FL	F	1989	NA	ES	*ATXN1*	30/38	29/37
I3	PD	F	1959	43	ES	*ATXN1*	29/38	28/37
I4	PD	M	1982	38	ES	*ATXN1*	29/37	28/36
I5	PNP	M	2006	9	ES	*ATXN1*	29/37	28/36
I6	MYO	F	1989	7	ES	*ATXN1*	27/37	26/36
I7	HSP	M	1973	44	ES	*ATXN1*	32/37	31/36
I8	Control	F	1963	-	GS	*ATXN1*	29/38	29/37
I9	ALS	F	NA	NA	GS	*ATXN1*	30/37	29/36
I10	Control	F	1964	-	GS	*ATXN1*	29/37	28/36
I11	ALS	F	1964	46	GS	*ATXN1*	29/37	28/36
I12	ALS	F	1949	67	GS	*ATXN1*	29/37	28/36
I13	ALS	M	1941	77	GS	*ATXN1*	30/37	30/37
I14	ALS	F	1966	48	GS	*ATXN1*	28/37	27/36
I15	ALS	M	1945	68	GS	*ATXN1*	29/37	28/36
I16	PD	M	1983	32	ES	*ATXN2*	22/32	22/33
I17	ataxia	M	1955	51	ES	*ATXN2*	22/31	22/32
I18	PD	F	1974	40	ES	*ATXN2*	22/30	22/31
I19	PNP	M	1999	14	ES	*ATXN2*	22/30	22/31
I20	PNP	F	1988	20	ES	*ATXN2*	22/30	22/31
I21	ataxia	F	1998	12	ES	*ATXN2*	22/30	22/31
I22	DYSL	M	1948	72	ES	*ATXN2*	22/30	22/31
I23	ataxia	F	2002	10	ES	*ATXN2*	22/30	22/31
I24	DMD	F	1984	15	ES	*ATXN2*	22/29	22/30
I25	ataxia	M	1948	50	ES	*ATXN2*	22/29	23/30
I26	ataxia	F	1968	NA	ES	*CACNA1A*	12/18	12/18
I27	ataxia	M	1997	19	ES	*CACNA1A*	11/18	11/18
I29	ataxia	M	1953	60	ES	*HTT*	21/37	21/38
I30	FTD & ALS	F	1962	59	ES	*HTT*	22/37	20/38
I31	PD	F	1966	36	ES	*HTT*	20/36	21/37
I32	ALS	M	1977	34	GS	*HTT*	19/38	20/39
I33	ALS	M	1980	38	GS	*HTT*	19/37	20/38
I34	ALS	M	1954	57	GS	*HTT*	19/37	20/38
I35	PD	M	1963	39	ES	*TBP*	37/45	37/43

Table S5. Thirty-four ALS patients carrying intermediate range (29-33 repeats) expansion at the *ATXN2* locus identified by ExpansionHunter and confirmed with conventional method.

ID	Gender	Date of Birth	Age on onset	NGS type	EH genotype	PCR-based
A1	F	1952	65	GS	22/32	22/33
A2	F	1951	66	GS	22/32	22/33
A3	M	1960	50	GS	22/32	22/33
A4	M	1956	58	GS	22/32	22/33
A5	M	1954	57	GS	22/32	22/32
A6	M	1950	66	GS	22/32	22/33
A7	F	1959	59	GS	22/32	22/33
A8	M	1962	52	GS	31/31	30/31
A9	M	1976	39	GS	22/31	22/32
A10	F	1947	55	GS	22/31	22/32
A11	F	1955	60	GS	22/31	22/32
A12	M	1965	49	GS	22/31	22/32
A13	M	1948	65	GS	22/31	22/31
A14	M	1931	81	GS	22/31	22/31
A15	M	1963	55	GS	22/31	22/32
A16	M	1980	31	GS	27/31	26/30
A17	M	1952	62	GS	18/30	18/30
A18	F	1975	41	GS	22/30	22/31
A19	M	1966	47	GS	22/30	22/31
A20	F	1936	77	GS	30/30	30/31
A21	M	1965	46	GS	22/30	22/31
A22	M	1948	69	GS	22/30	22/31
A23	M	1949	61	GS	22/30	22/30
A24	M	1956	55	GS	22/30	22/31
A25	M	1966	47	GS	22/29	23/30
A26	F	1963	52	GS	22/29	22/30
A27	M	1966	48	GS	22/29	23/30
A28	F	1957	58	ES	22/31	23/32
A29	M	1960	60	ES	22/31	22/32
A30	F	1954	68	ES	22/30	22/31
A31	M	1960	54	ES	22/30	22/31
A32	M	1970	44	ES	30/30	25/31
A33	M	1955	64	ES	26/29	27/30
A34	M	1964	57	ES	22/29	23/30

Table S6. Gene-level summary of pathogenic, intermediate, normal, and false-positive calls in 113 individuals for whom both alleles were evaluated by ExpansionHunter and PCR.

Gene	Pathogenic	Intermediate	Normal	False Positive
AR	1	0		1
ATN1	4	0	4	0
ATXN1	4	15	20	1
ATXN2	8	44	52	0
CACNA1A	1	2	3	0
HTT	14	6	20	0
PABPN1	4	0	4	0
TBP	6	1	8	1

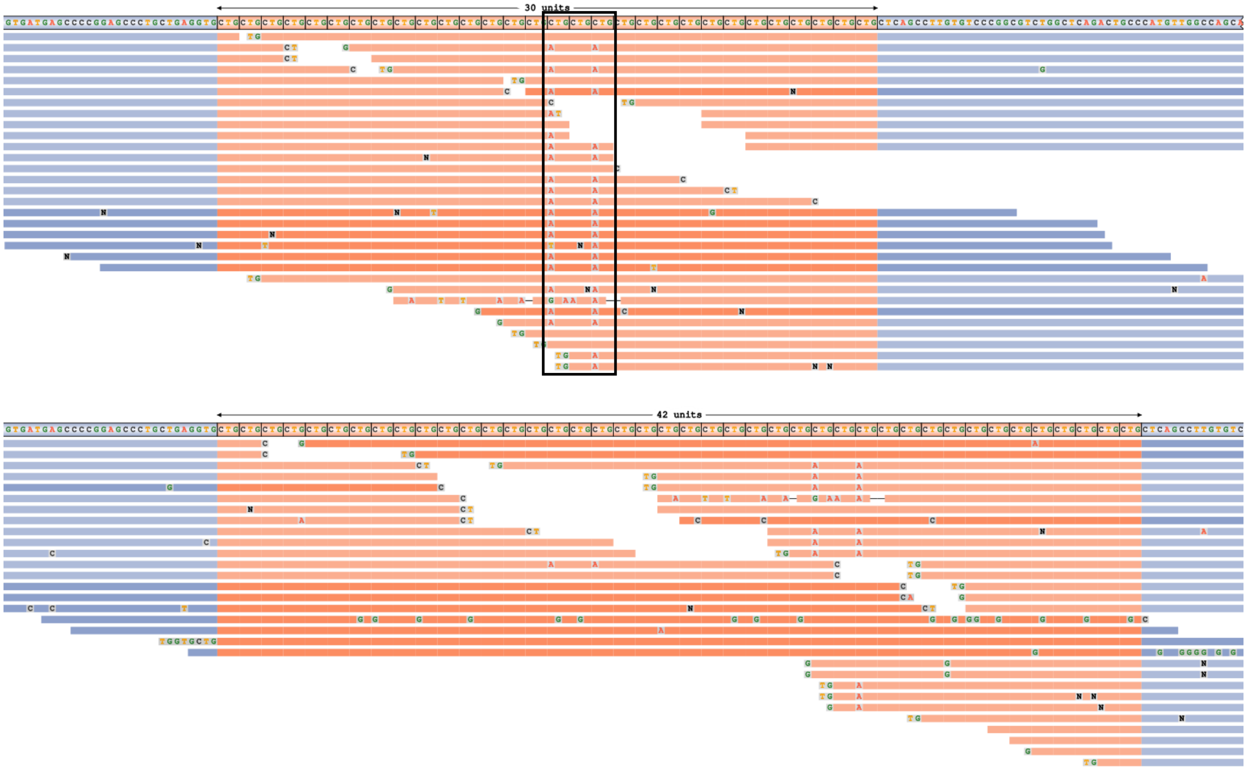

Figure S8. REViewer visualization of the ***ATXN1*** repeat locus in F04-P1. The shorter allele has two histidine interruptions, while the expanded allele lacks.

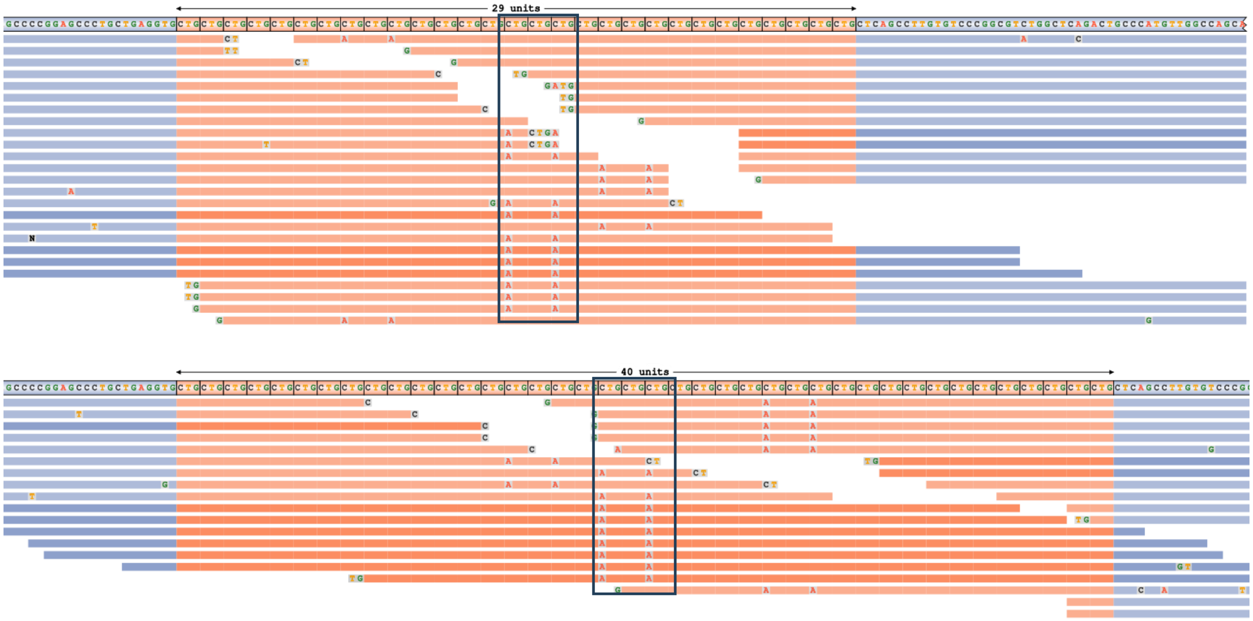

Figure S9. REViewer visualization of the ***ATXN1*** repeat locus in F05-P1. Both alleles have two histidine interruptions.

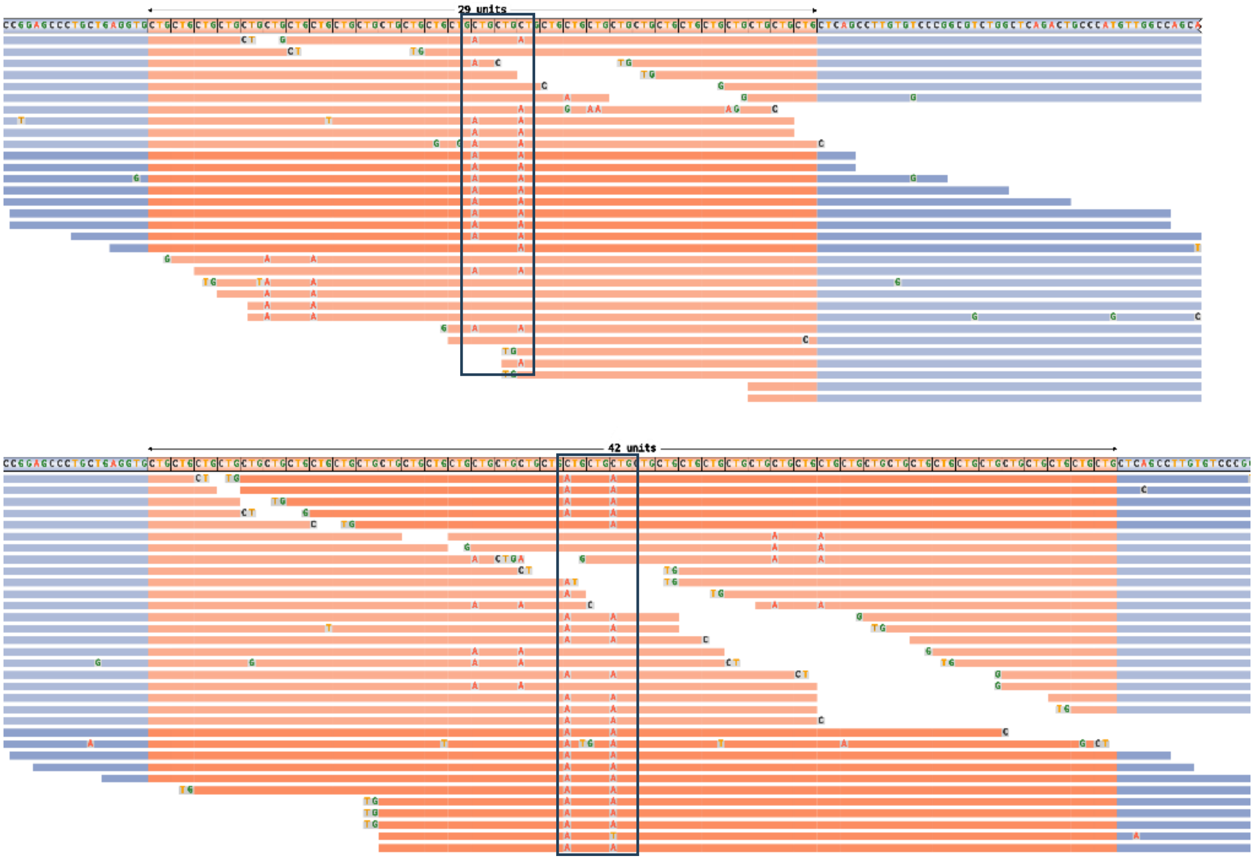

Figure S10. REViewer visualization of the ***ATXN1*** repeat locus F06-P1. Both alleles have two histidine interruptions.

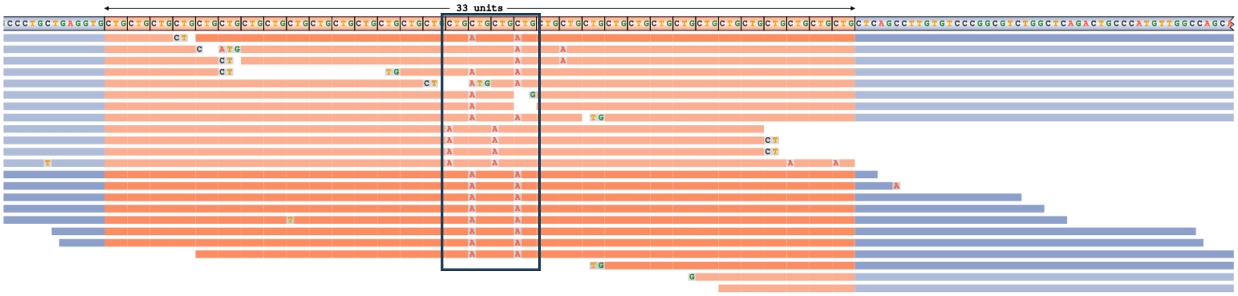

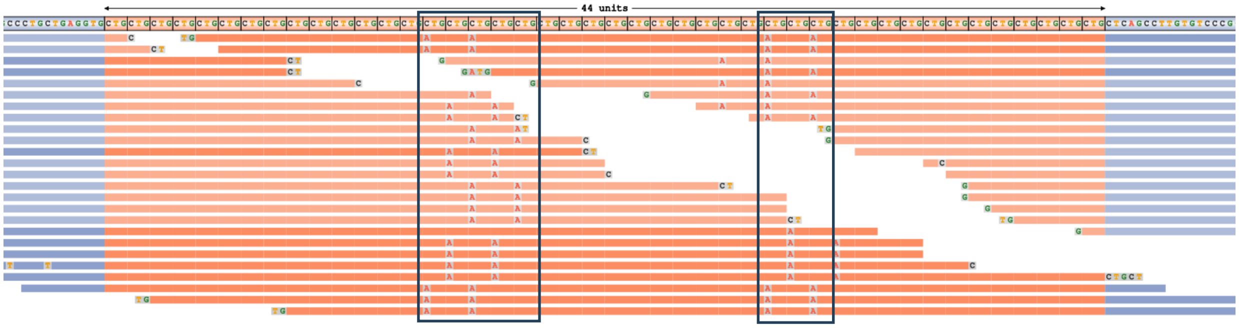

Figure S11. REViewer visualization of the ***ATXN1*** repeat locus in F03-P1. Shorter allele has two histidine, while the expanded allele has four histidine.

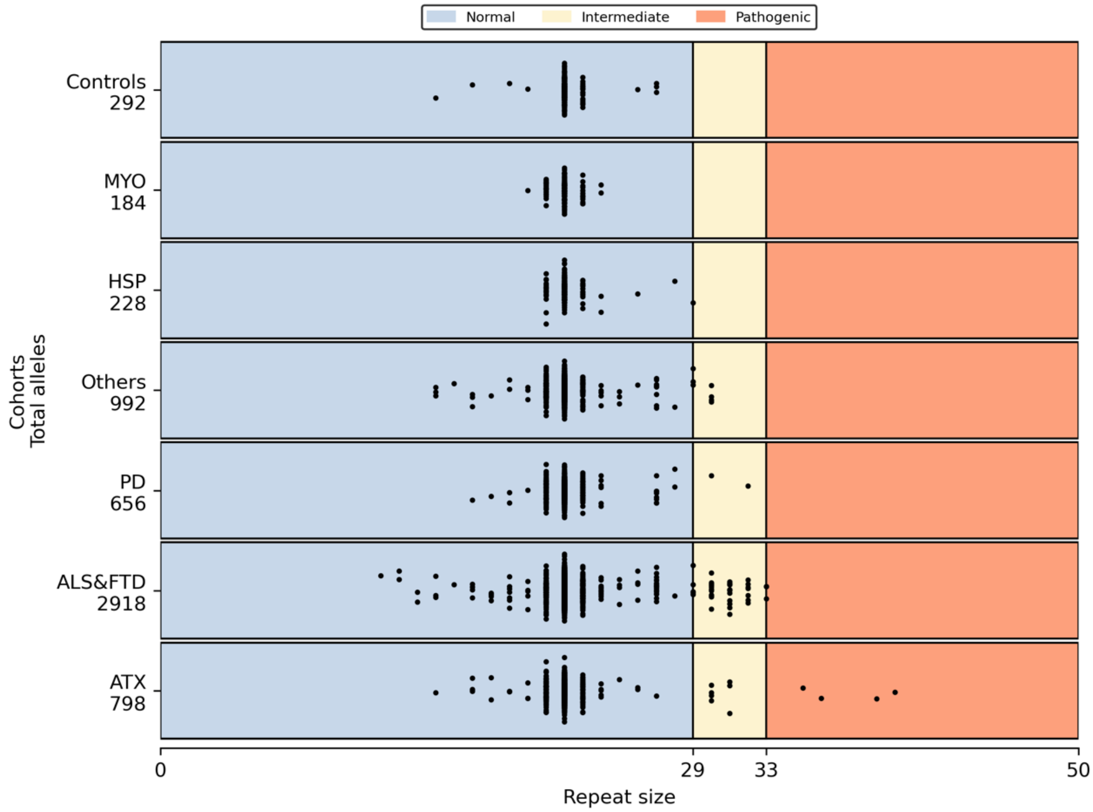

Figure S12. Distribution of *ATXN2* repeat size genotyped by EH across disease and control cohorts. The total number of alleles in each cohort, corresponding to twice the sample size, is given below the cohort's label.

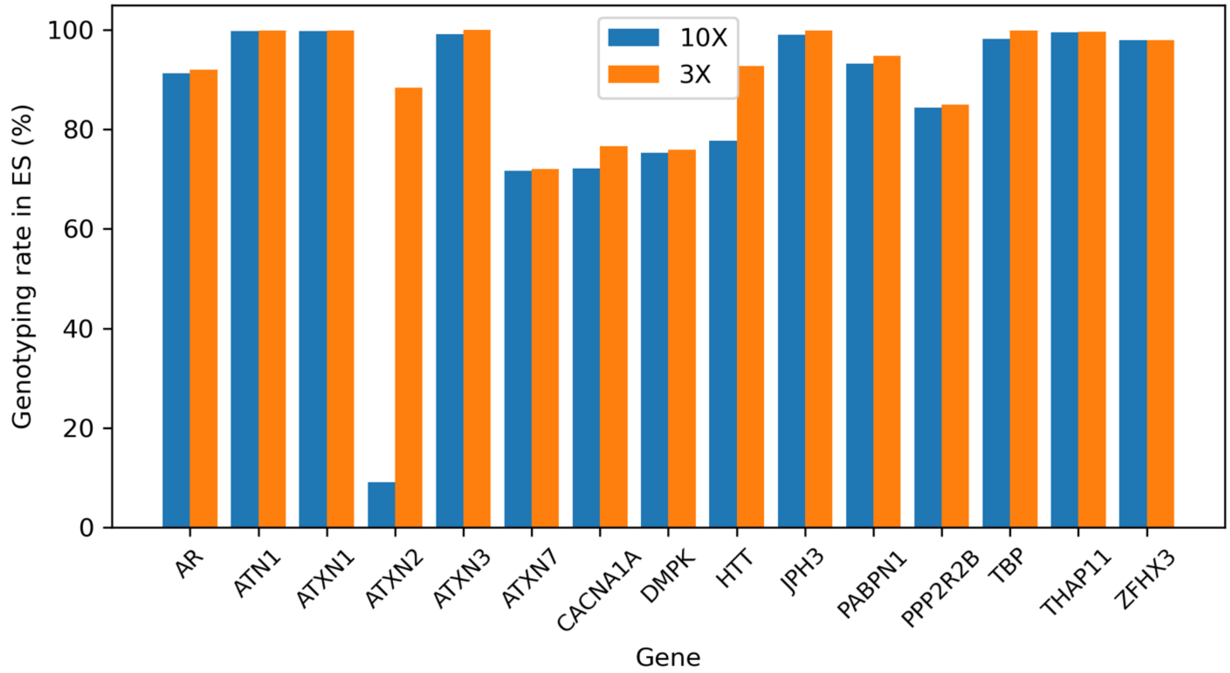

Figure S13. Genotyping rates across 15 STR loci in exome data using default (10×) and reduced (3×) coverage thresholds in ExpansionHunter. The default setting requires a minimum of 10× coverage for genotyping.

Table S7. Demographic characteristics of the control cohort (n = 146). Control individuals were recruited between 2013 and 2017.

Birth year	Total	Male	Female	% of total
≤1950	25	11	14	17.1
1951–1970	73	34	39	50
>1970	48	24	24	32.9
